# The influence of exposure to immunosuppressive treatment during pregnancy on renal function and rate of apoptosis in native kidneys of female Wistar rats

**DOI:** 10.1007/s10495-016-1281-y

**Published:** 2016-09-01

**Authors:** Joanna Kabat-Koperska, Agnieszka Kolasa-Wołosiuk, Irena Baranowska-Bosiacka, Krzysztof Safranow, Danuta Kosik-Bogacka, Izabela Gutowska, Anna Pilutin, Edyta Gołembiewska, Karolina Kędzierska, Kazimierz Ciechanowski

**Affiliations:** 1 Department of Nephrology, Transplantology and Internal Medicine, Pomeranian Medical University, Powstancow Wielkopolskich 72, 70-111 Szczecin, Poland; 2 Department of Histology and Embryology, Pomeranian Medical University, Powstancow Wielkopolskich 72, 70-111 Szczecin, Poland; 3 Department of Biochemistry and Medical Chemistry, Pomeranian Medical University, Powstancow Wielkopolskich 72, 70-111 Szczecin, Poland; 4 Department of Biology and Medical Parasitology, Pomeranian Medical University, Powstancow Wielkopolskich 72, 70-111 Szczecin, Poland; 5 Department of Biochemistry and Human Nutrition, Pomeranian Medical University, Broniewskiego 24, 71-460 Szczecin, Poland

**Keywords:** Immunosuppressive drugs, Kidney, Pregnancy, Transplantation, Wistar rats

## Abstract

Pregnancy puts a significant additional strain on kidneys. The aim of our study was to investigate the impact of immunosuppressive drugs on changes in native kidneys in female Wistar rats after exposure during pregnancy. The study was conducted on 32 dams, subjected to immunosuppressive regimens commonly used in the therapy of human kidney transplant recipients (cyclosporine A, mycophenolate mofetil and prednisone; tacrolimus, mycophenolate mofetil and prednisone; cyclosporine A, everolimus and prednisone). The animals received drugs for 2 weeks before pregnancy and during 3 weeks of pregnancy. In all treated dams lower body weight (but not kidney mass) and alterations in serum sodium and chloride ions were found; serum creatinine concentration was increased in dams treated with cyclosporine A, everolimus and prednisone. All treatment groups of dams showed increased apoptosis in the distal tubules. In histological examination the changed intensity of acidophilic or basophilic cytoplasm of epithelial cells was found in kidneys of rats treated with calcineurin inhibitors, mycophenolate mofetil and prednisone. All immunosuppressive regimens caused abnormalities affecting nephron tubules. Regimens containing calcineurin inhibitors and mycophenolate mofetil caused higher rate of apoptosis and more pronounced histopathological changes. Regimen based on everolimus despite the lower rate of apoptosis in the proximal tubules and lower accumulation of kidney injury markers revealed higher serum creatinine concentration. Thus, interpretation which combination of drugs is better or worse for long-lasting functioning of kidneys in pregnant females requires further studies.

## Introduction

Female kidney graft recipients in reproductive age recover fertility 6 months after successful renal transplantation. Pregnancy does not appear to adversely affect graft function, when this function is stable prior to pregnancy. While the shortest safe interval from transplant to conception has not been established, 1–2 years are reasonable milestones [1]. These pregnancies are high-risk. Some immunosuppressive drugs are considered to be relatively safe during pregnancy (cyclosporine A, CsA; tacrolimus, Tc; azathioprine and steroids) while others are contraindicated because of toxicity (mycophenolate mofetil, MMF and mammalian target of rapamycin, mTOR inhibitors). However, experience regarding use of many immunosuppressive drugs in human pregnancy is limited. The transplanted organ or native kidneys in healthy humans must adapt to additional strain associated with pregnancy. Renal function undergoes several physiological adaptations- renal perfusion and renal blood flow increases by 80 % above nonpregnant values. It leads to a rise in the glomerular filtration rate (GFR) that persists until term and translates to a fall in various serum markers of renal clearance, including creatinine, urea, uric acid [15]. Previous studies were focused mainly on the effects of prenatal immunosuppression on rat renal development in the offspring [17, 18, 20]. The aim of our study was to investigate the impact of immunosuppressive drugs considered to be acceptable during pregnancy and those contraindicated during pregnancy together in combinations on changes in native kidneys in female Wistar rats exposed to such drugs during pregnancy.

## Materials and methods

### Animals and treatment

The study was conducted on 32 female and 8 male Wistar rats (the Centre of Experimental Medicine, Medical University in Bialystok, Poland). At the start of the experiment, the rats were 12 weeks old and their mean weight was 230 g. The animals had genetic and health certificates issued by a veterinarian. This study was approved by the Local Ethical Committee for Experiments on Animals in Szczecin (No. 12/2013, dated 24 Oct 2013). All procedures performed in studies involving animals were in accordance with the ethical standards of the institution or practice at which the studies were conducted. The animals were housed singly, kept on a 12-hour-light–dark cycle and were given feed Labofeed H (Morawski, Kcynia, Poland) and water ad libitum.

The experiment was performed using the pharmaceutical form of each drug. The animals received drugs by oral gavage (at a dose volume of 5 ml/kg daily). The doses used in the study were as follows: tacrolimus (Prograf, Astellas): 4 mg/kg/day; mycophenolate mofetil (CellCept, Roche): 20 mg/kg/day; cyclosporin A (Sandimmun Neoral, Novartis): 5 mg/kg/day; everolimus (Certican, Novartis): 0.5 mg/kg/day and prednisone (Encorton, Polfa): 4 mg/kg/day. The drug doses were based on data available in the literature [6, 7, 12, 14, 19, 22] to reach the level within a therapeutic range. A diagram of the study is presented in Table 1. The animals received medication every 24 h for approximately 5 weeks (2 weeks after the acclimatization period prior to mating-when placed with males 1:1 in separate cages—and later after mating during 3 weeks of pregnancy). After mating each pregnant female rat was housed in a separate cage. Once a week the animals were weighed again, and medication dose was adequately adjusted based on the changed weight. After delivery the treatment was stopped (no drugs administration during lactation period as in humans breastfeeding is not advised while taking immunosuppressive drugs). 31 female rats completed the study and 69 pups from control group, 13 from CMG group and only 1 pup from CEG group were born. The dams were sacrificed at weaning (day 21 after delivery—we decided not to euthanize them earlier as their offspring had to stay alive for other studies).

**Table 1:** The study model

Group	Glucocortico-steroids (G)	Tacrolimus (T)	Cyclosporin A (C)	Everolimus (E)	Mycophenolate mofetil (M)
Control group (n = 7)	**−**	**−**	**−**	**−**	**−**
CMG group (n = 8)	**+**	**−**	**+**	**−**	**+**
TMG group (n = 8)	**+**	**+**	**−**	**−**	**+**
CEG group (n = 8)	**+**	**−**	**+**	**+**	**−**

Abbreviations of the drugs that are used to name the study groups in brackets

CMG—CsA + MMF + prednisone; TMG—Tc + MMF + prednisone; CEG—CsA + everolimus + prednisone

The female rats were euthanized by penthobarbitalum sodium (Polpharma) injection administered intraperitoneally at 40 mg/kg body weight. Their body weight was measured. Blood samples were obtained for lab tests (sodium, potassium, chloride, urea, creatinine, uric acid, total protein and albumin serum concentrations). Subsequently, necropsies of all rats were performed, the collected kidneys were weighed. The left kidney was fixed in 4 % buffered formalin solution for histological examination. The right kidney was placed in liquid nitrogen and then stored at −80  C for markers of kidney injury analysis.

### Markers of kidney injury

Kidney injury molecule KIM-1 (TIM-1), monocyte chemoattractant protein 1(MCP-1) and neutrophil-gelatinase associated lipocalin (NGAL) were assessed in homogenized renal tissue of female rats.

#### Homogenization protocol

Frozen whole kidneys were taken from liquid nitrogen and placed in a thermobox (−21 °C). A small fragment of the tissues was placed in a metal homogenizator (previously cooled in a container with liquid nitrogen) and poured on 2–3 times with liquid nitrogen; then it was fragmented with a few hammer blows (4–5 times) against a metal mandrel (also previously cooled in a container with liquid nitrogen). Pulverized and frozen sample (volume equal to a approximately 1 mg of protein) was placed with a cooled spoon in an Eppendorf tube containing 500 µL of appropriate buffer (according to commercial enzyme assay kit procedure) previously cooled to the temperature of 4 °C. After a short vortexation, homogenization was carried out with a knife homogenizator for about 15 s. Extract mixtures were centrifuged (3000 g for 10 min, at 4 °C) and the supernatants stored at −80 °C and used for enzyme assays.

#### KIM-1 (TIM-1)

*KIM*-*1 (TIM*-*1)* was assessed using the Quantikine Rat TIM-1/KIM-1/HAVCR Immunoassay (R&D System, USA). *MCP*-*1* was assessed using the rat MCP-1 Instant ELISA (an enzyme-linked immunosorbent assay for the quantitative detection of rat MCP-1, eBioscience, An Affymetrix Company, Vienna, Austria). *NGAL* was assessed using the rat NGAL/lipocalin2/oncogene24p3 ELISA (Wuhan EIAab Science Company, Wuhan, China).

#### Protein concentration measurement

All concentrations of markers of kidney injury were expressed as pg per 1 mg of protein. To determine the protein content in the sample The Micro BCA Protein Assay Kit (Thermo Scientific, Pierce Biotechnology, USA) was used according to the manufacturer’s protocol. This Kit is a detergent-compatible bicinchoninic acid formulation for the colorimetric quantitation of total protein [28].

### Histological evaluation and its criteria

Paraffin slides (3 µm) were stained with hematoxylin-eosin (H&E) and underwent general histological examination. The thickness of renal cortex and diameter of glomeruli in kidneys were measured. The samples were independently examined by two experienced pathologists.

### Apoptosis assessment

**Fig. 1:**
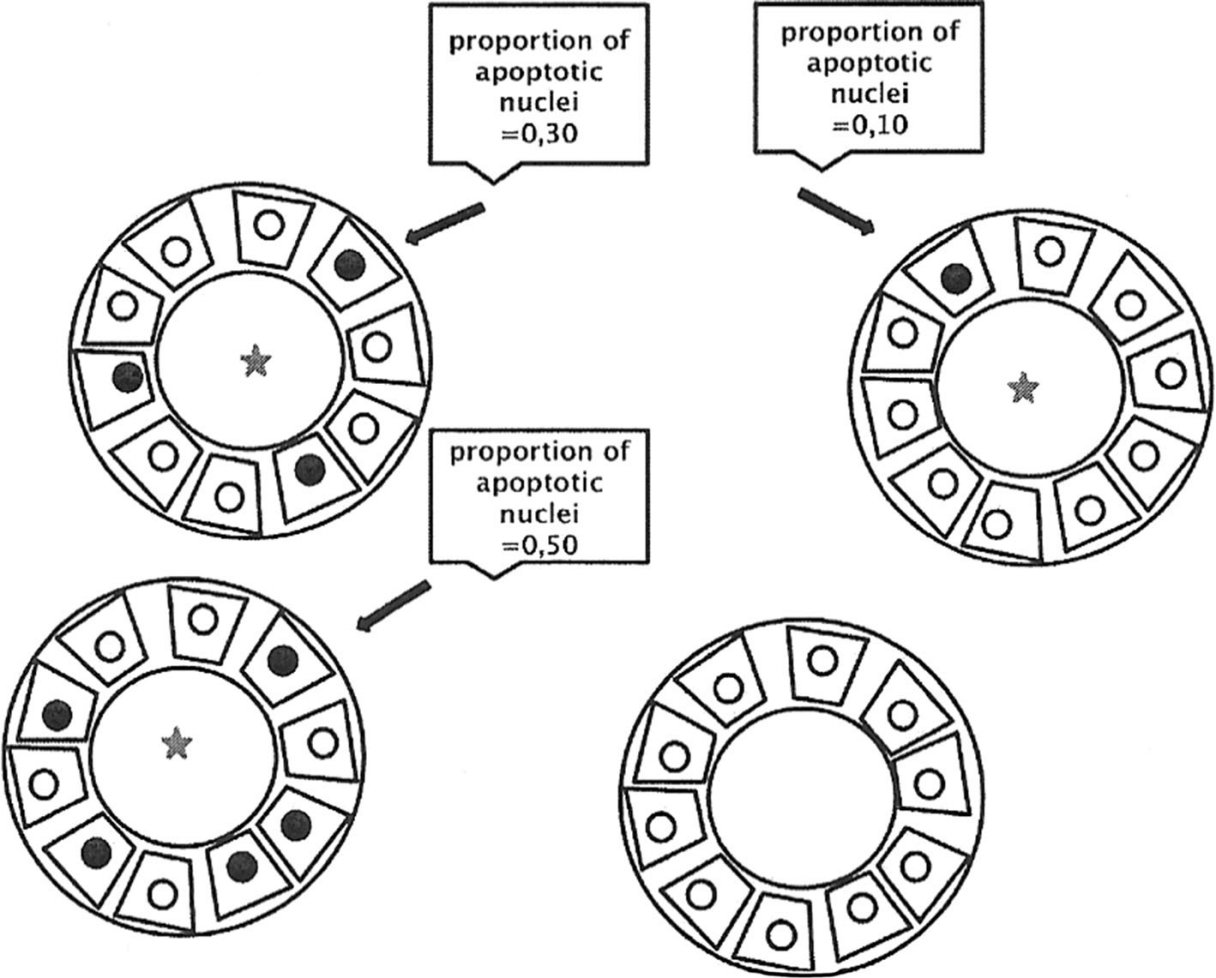
Renal tubules cross sectional structures with a positive staining to detect apoptosis (*marked with a star*). Calculation method for percentage of apoptotic nuclei in each tubule

In order to evaluate tubular cell apoptosis, the TUNEL reaction was performed (terminal deoxynucleotidyl transferase-mediated deoxyuridine triphosphate biotin nick-labeling). An important feature of apoptotic cells is the fragmentation of DNA into pieces, whose length is equal to a multiple nucleosome length (180–200 bp), reflecting the structure of the histone octamers. The reaction product was assessed by light microscopy. This test was considered positive in the presence of colour reaction in at least one nucleus in the tubule. Positive results are expressed as a percentage of proximal or distal tubules with the stained nucleus. The number of apoptotic nuclei were analysed at each proximal and distal tubule in the samples separately (Fig. 1).

### Drug concentration in blood

For the evaluation of drug concentrations in rats’ blood we used a separate group of pregnant female rats (n = 14) at the corresponding age. These rats were given identical doses of the drugs by oral gavage (which were adjusted based on weight). The drug concentration was determined in accordance with the literature [14, 23] after 4 h of oral administration—an optimal time for determining the concentration of drugs in blood, due to different drug metabolism in rats compared to humans. The concentration of drugs in blood of all the rats was determined after 1 week of taking drugs once daily from the time of first administration. The concentration of CsA was determined with Abbott AxSYM assay (fluorescence polarisation immunoassay—FPIA). To determine Tc level we used IMx assay (Microparticle Enzyme Immunoassay—MEIA). The test was performed using an Abbott analyser (Abbott Laboratories, Park, USA). The study was carried out at the Clinical Central Laboratory in Szczecin. The concentration of everolimus was determined at the Laboratory of Mass Spectometry IBB PAN in Warsaw using original author’s method (ultra performance liquid chromatography/tandem mass spectrometry UPLC/MS/MS—[27].

### Statistical analysis

The values of quantitative variables were compared between groups using non-parametric tests (Kruskal–Wallis and Mann–Whitney U test), due to most of the data being not normally distributed (as assessed by Shapiro–Wilk’s test). The mean, standard deviation, median, minimum and maximum values were calculated for each group. The cut-off level of statistical significance was set at p < 0.05. Calculations were performed using Statistica 12 software.

## Results

**Fig. 2:**
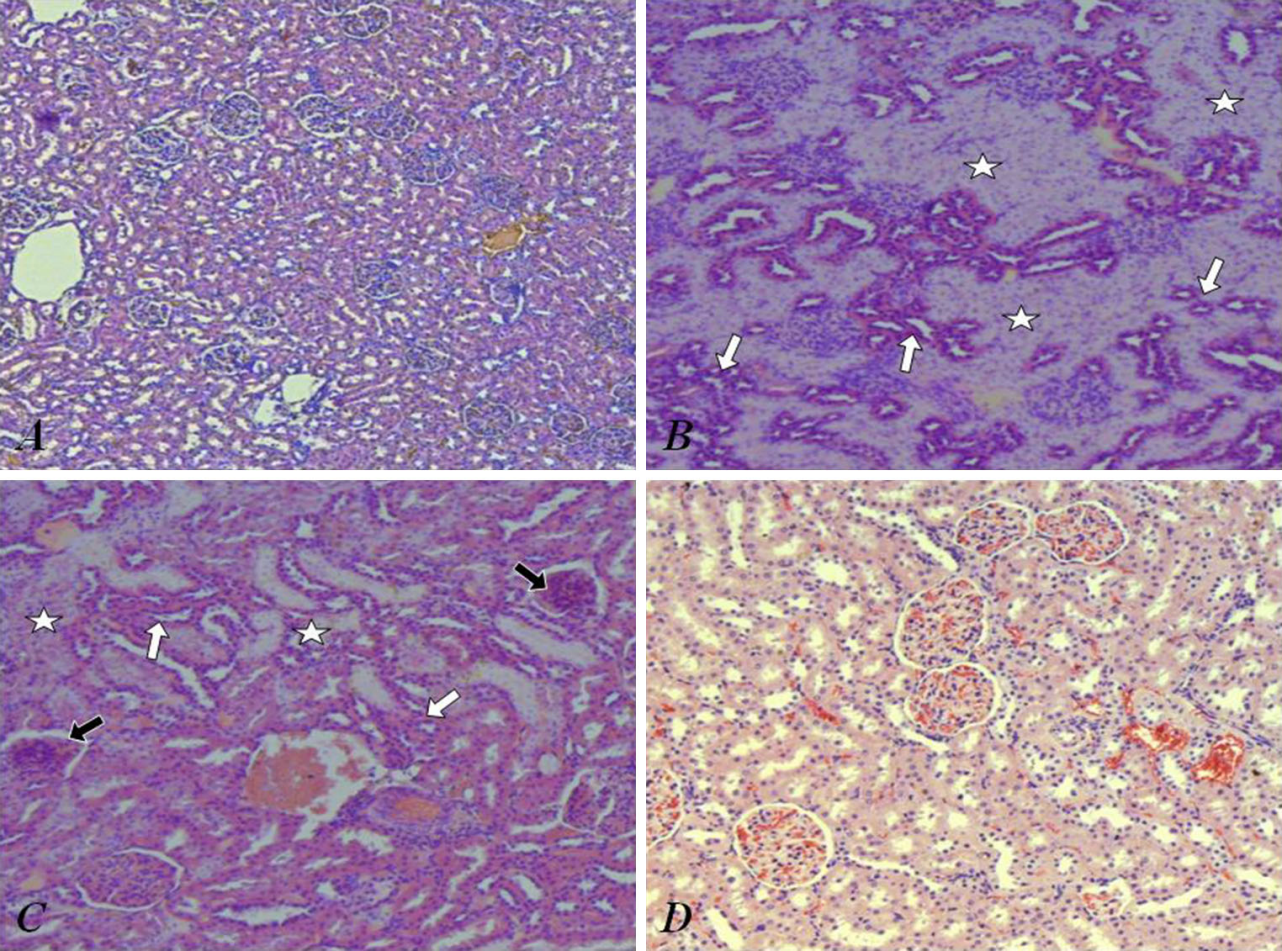
Image of kidney from control rat (**a**) and rats from treatment groups (**b**–**d**). Visible darker staining (more acidophilic) distal convoluted tubules (*white arrows*) and lighter staining proximal convoluted tubules (*white asterisks*) in kidney of cyclosporine + MMF + prednisone (**b**) and tacrolimus + MMF + prednisone (**c**) treated rats unlike in control rat (**a**). The acidophilic proximal and basophilic distal convoluted tubules within the kidney from everolimus + cyclosporine + prednisone treated rat (**d**) like in control rat (**a**). The bloodshot of renal parenchyma of everolimus + cyclosporine + prednisone treated rat (**d**) similar but not so intense like in tacrolimus + MMF + prednisone treated rat (**c**). A few collapsed renal glomeruli (*black arrows*) in rat from the latter group (**c**). Objective magnification: A x10, B x20, C x20, D ×40. Staining:H&E

The results of the research and statistical analysis are presented in Tables 2, 3, 4, 5, 6 and Fig. 2. The drug concentrations in blood are shown in Table 7.

**Table 2:** Body and kidney weight of female rats in control and treatment groups

Parameter/group (g)		Control group (n = 7)	CMG group (n = 8)	TMG group (n = 8)	CEG group (n = 8)	p (Kruskal–Wallis test)
Body mass (g)	AM ± SD	274.97 ± 14.96	244.185 ± 26.36	253.87 ± 19.96	253.98 ± 14.92	**0.033**
	Median	280	233.71*	253.2*	256.99*	
	Range	250–288.64	224.72–303.52	223.66–287.2	236.66–273.76	
Kidney mass (g)	AM ± SD	0.9 ± 0.08	0.8 ± 0.11	0.85 ± 0.15	0.80 ± 0.06	0.106 (NS)
	Median	0.87	0.78	0.82	0.81	
	Range	0.8–1.02	0.64–1.02	0.66–1.3	5.46–6.05	

Bold value indicates statistical significance at p < 0.05

*AM* arithmetic mean; *SD* standard deviation; *p* level of significance; *NS* difference non-significant; CMG—CsA + MMF + prednisone; TMG—Tc + MMF + prednisone; CEG—CsA + everolimus + prednisone

* p < 0.05 vs control group (Mann–Whitney test)

**Table 3:** Biochemical serum test results of female rats in control and treatment groups

Parameter/group		Control group (n = 7)	CMG group (n = 8)	TMG group (n = 8)	CEG group (n = 8)	p (Kruskal–Wallis test)
Sodium (mmol/L)	AM ± SD	144.83 ± 1.17	141.83 ± 1.72	144.5 ± 2.2	148.86 ± 1.95	**0.0006**
	Median	145	141.5*	144	149**	
	Range	143–146	140–145	141–148	146–152	
Potassium (mmol/L)	AM ± SD	4.45 ± 0.49	4.63 ± 0.47	5.5 ± 1.09	5.9 ± 0.89	0.1061 (NS)
	Median	4.45	4.6	5.75	5.6	
	Range	4.1–6.4	4.1–5.4	3.8–6.6	4.9–7.1	
Chloride (mmol/L)	AM ± SD	99 ± 3.03	101 ± 3.22	102.25 ± 1.16	103.57 ± 2.37	**0.03**
	Median	100	100.5	102*	104*	
	Range	94–102	98–106	100–104	101–108	
Total protein (g/L)	AM ± SD	66.67 ± 5.16	72.5 ± 10.09	73.625 ± 3.46	71.43 ± 3.505	0.0785 (NS)
	Median	68	69.5	72.5	72	
	Range	60–73	63–92	70–80	66–75	
Albumin (g/L)	AM ± SD	31.33 ± 2.73	33.17 ± 6.37	32.625 ± 3.7	35.285 ± 1.7	0.1209 (NS)
	Median	31.5	31.5	33	36	
	Range	28–35	28–45	25–37	33–37	
Creatinine (mg/dL)	AM ± SD	0.555 ± 0.07	0.52 ± 0.05	0.6 ± 0.07	0.65 ± 0.06	**0.0107**
	Median	0.545	0.515	0.585	0.66**	
	Range	0.48–0.64	0.48–0.61	0.51–0.69	0.52–0.7	
Urea (mg/dL)	AM ± SD	52.33 ± 6.18	51 ± 2.28	46 ± 10.555	54.71 ± 4.75	0.1428 (NS)
	Median	52	51	47.5	57	
	Range	43–60	47–54	26–62	46–60	
Uric acid (mg/dL)	AM ± SD	4.68 ± 3.33	3.5 ± 1.685	5.84 ± 2.38	5.87 ± 2.46	0.3176 (NS)
	Median	4.35	3.45	5.95	6.4	
	Range	1.5–8.9	1.4–6.4	2.9–9.3	1.6–8.1	

Bold values indicate statistical significance at p < 0.05

*AM* arithmetic mean; *SD* standard deviation; *p* level of significance; *NS* difference non-significant; CMGCsA + MMF + prednisone; TMG—Tc + MMF + prednisone; CEG—CsA + everolimus + prednisone

* p < 0.05, ** p < 0.001 vs control group (Mann–Whitney test)

**Table 4:** Concentrations of renal injury markers in kidney of female rats in control and treatment groups

Parameter/group		Control group (n = 7)	CMG group (n = 8)	TMG group (n = 8)	CEG group (n = 8)	p (Kruskal–Wallis test)
TIM-1 (pg/mg protein)	AM ± SD	89,55 ± 30,74	87,95 ± 33,66	95,44 ± 49,24	61,08 ± 20,71	0.30 (NS)
	Median	96,59	96,43	101,135	54,01	
	Range	37,91–116,73	51,85–134,44	29,8–174	38,02–88,59	
MCP–1 (pg/mg protein)	AM ± SD	24,27 ± 11,19	21,66 ± 33,01	20,13 ± 11,21	13,7 ± 9,08	0.17 (NS)
	Median	26,09	9,74	18,51	15,46	
	Range	11,21–41,755	1,82–94,56	6,63–41,49	0,54–25,78	
NGAL (pg/mg protein)	AM ± SD	131,92 ± 18,38	142,24 ± 32,35	149,58 ± 43,465	108,23 ± 21,26	0.066*
	Median	137,65	135,35^	154,32^	109,33	
	Range	106,81–158	81,68–178,285	100,33–236,38	79,72–139,79	

*AM* arithmetic mean; *SD* standard deviation; *p* level of significance; *NS* difference non-significant; CMG—CsA + MMF + prednisone; TMG—Tc + MMF + prednisone; CEG—CsA + everolimus + prednisone

* p = 0.049 for difference between three treatment groups (Kruskal–Wallis test)

^ p < 0.05 vs CEG group (Mann–Whitney test)

**Table 5:** Apoptosis intensity in renal cortex of female rats in control and treatment groups

IHC reaction/group( %)		Control group (n = 7)	CMG group (n = 8)	TMG group (n = 8)	CEG group (n = 8)	p (Kruskal–Wallis test)
Apoptosis in proximal tubules (%)	AM ± SD	4.69 ± 2.56	11.95 ± 7.2	20.40 ± 7.84	3.035 ± 1.05	**0.0011**
	Median	4.34	10.35*	19***	2.975	
	Range	1.0–8.15	5.21–21.89	12.275–29.435	2.07–4.12	
Apoptosis in distal tubules (%)	AM ± SD	15.93 ± 11.01	88.13 ± 7.57	61.105 ± 8.22	44.9 ± 14.45	**0.0004**
	Median	17.38	88.13**	60.74***	40.835**	
	Range	3.125–32.3	82.14–92.41	47.79–75.2	32.67–65.25	

Bold values indicate statistical significance at p < 0.05

*AM* arithmetic mean; *SD* standard deviation; *p* level of significance; *NS* difference non-significant; CMG—CsA + MMF + prednisone; TMG—Tc + MMF + prednisone; CEG—CsA + everolimus + prednisone

* p < 0.05, ** p < 0.01, *** p < 0.001 vs control group (Mann–Whitney test)

**Table 6:** Thickness of renal cortex and diameter of glomeruli of female rats in control and treatment groups

Measurement/group (µm)	Control group (n = 7)	CMG group (n = 8)	TMG group (n = 8)	CEG group (n = 8)	p (Kruskal–Wallis test)
Thickness of cortex	972.86 ± 135.67	881.41 ± 166.265	837.39 ± 110.31	984.62 ± 69.07	0.14 (NS)
Diameter of glomeruli	68.30 ± 4.34	64.35 ± 7.08	66.18 ± 5.65	72.28 ± 6.97	0.25 (NS)

Results are presented as arithmetic mean ± standard deviation

*P* level of significance; *NS* difference non-significant; CMG—CsA + MMF + prednisone; TMG—Tc + MMF + prednisone; CEG—CsA + everolimus + prednisone

**Table 7:** The medication concentration and weight of female rats in additional control and treatment groups

	Control group (n = 3)	CMG group (n = 3)	TMG group (n = 4)	CEG group (n = 4)
Cyclosporin A (ng/mL)	–	69.37 ± 45.61	–	50.35 ± 8.80
Tacrolimus (ng/mL)	–	–	7.00 ± 6.61	–
Everolimus (ng/mL)	–	–	–	1.43 ± 0.17
Body mass (g)	260.00 ± 16.00	240.00 ± 21.00	255.00 ± 12.50	245.00 ± 22.50

Results are presented as arithmetic mean ± standard deviation

CMG—CsA + MMF + prednisone; TMG—Tc + MMF + prednisone; CEG—CsA + everolimus + prednisone

### Body and kidney weight

We found lower body weight of dams from treatment groups as compared to control dams. Although these dams reached lower body weight, the mass of their kidneys was not significantly reduced (Table 2). The kidney/body weight ratio was 0.003 for dams from control group and all treatment groups.

### Laboratory blood test results

We found lower value of serum sodium concentration in dams from CMG group and higher value of serum sodium concentration in dams from CEG group in comparison to control group. We also observed higher value of serum chloride concentration in dams from TMG and CEG group. Serum creatinine concentration was increased in dams from CEG group (Table 3).

### Markers of kidney injury

We found no statistically significant differences in renal tissue concentration of KIM-1 (TIM-1), MCP-1 and NGAL between control group and all treatment groups (Table 4). We observed higher levels of NGAL in dams from CMG and TMG group in comparison to dams from CEG group (p = 0.040 and p = 0.038, respectively, Mann–Whitney U test).

### Apoptosis assessment

Apoptosis was evaluated in proximal and distal tubules of renal cortex. CMG and TMG groups of dams exhibited more pronounced apoptosis in the proximal nephron tubules compared to control and CEG group (apoptosis intensity in proximal tubules was similar in control and CEG group). All treatment groups of dams showed much more pronounced apoptosis in the distal tubules compared to control group (Table 5).

### Histopathological evaluation

The arrangement of individual elements in renal parenchyma of control rat (Fig. 2a) and rats from treatment groups (Fig. 2b–d) was unchanged: renal cortex contained renal glomeruli enveloped by proximal and distal convoluted tubules (PCT and DCT, respectively). However, tissues of rats treated with immunosuppressive drugs differed from control tissues: PCT had lighter staining and DCT was much more acidophilic in kidneys of rats from CMG group (Fig. 2b) and TMG group (Fig. 2c). The more acidophilic PCT and more basophilic DCT were present in kidneys of rats from CEG group (Fig. 2d) similarly to control rats (Fig. 2a). Observed locally, congested and dilated small blood vessels and capillaries in renal cortex of rats from TMG (Fig. 2c) and CEG group (Fig. 2d) had resulted in bloodshot-like image of renal parenchyma. Moreover, a few collapsed glomeruli were visible within the cortex of rats from TMG group (Fig. 2c). The measurements of thickness of renal cortex and diameter of glomeruli in all groups showed no statistically significant differences (Table 6).

## Discussion

In our study models of treatment comparable to immunosuppressive therapy commonly used in clinical practice in humans were attempted. In female rats before pregnancy allograft kidney transplantation was not performed—we analysed morphology and function of native kidneys. We wanted to exclude factors confounding the direct impact of immunosuppressive drugs on the structure and function of kidney like ischemia–reperfusion damage, humoral and cellular rejection, the quality of the harvested organ etc.

In dams from treatment groups we noticed a reduction in body weight but mass of kidney was not decreased. A reduction in body weight in rats treated with immunosuppressive drugs was not surprising as confirmed previously in several studies [9, 10, 21], tacrolimus and mTOR inhibitor, rapamycin had the strongest negative influence on the rat body weight. MMF had no significant effect on animal body weight.

Analysing biochemical parameters we found some changes in concentrations of ions (in CMG group- lower serum sodium concentration; in TMG group- higher serum chloride concentration; in CEG group- higher serum sodium and chloride concentration). An exposure to combination of immunosuppressive drugs could influence and change the transport of ions in nephrons. Esteva-Font et al. [3] found an increase in the Na–K-2Cl co-transporter of the loop of Henle (NKCC2) in CsA-treated rats. Cui et al. [2] identified two genes, Slc12a3 and kidney-specific Wnk1 (KS-Wnk1), that could potentially be involved in the mechanism of calcineurin inhibitors induced nephrotoxicity. They found down-regulation of these genes in animals treated with CsA or Tc and this decreased expression could have altered the sodium chloride reabsorption and sodium transport in the distal tubules. Therefore the changes in concentrations of ions in our experiment could be explained by influence of different combinations of immunosuppressive drugs on the function of ions transporters.

Serum creatinine concentration was increased only in dams from CEG group and comparable to control in CMG and TMG groups. It is consistent with previous observations [9]—as rats treated with MMF had lower creatinine serum concentrations compared to rats not treated with this drug [10]. This association was found in previous studies, not only in rats [26], but as well in humans [4]. Moreover, in study of Piao et al. [19] where combination of everolimus and CsA was used, everolimus aggravated CsA-induced nephrotoxicity.

Recently biomarkers reflecting kidney injury prior to the elevation of serum creatinine concentration were identified—TIM-1, also known as KIM-1, MCP-1 and NGAL protein. We found no statistically significant differences in the concentration of renal injury markers between control group and all treatment groups, but we observed higher levels of NGAL in dams from CMG and TMG group in comparison to dams from CEG group. Data from previous studies revealed increased expression of KIM-1 in rat kidney in a model of cyclosporine-induced nephrotoxicity [5, 24]. Tacrolimus up-regulated renal cortical gene for MCP-1 [25]. On the other hand, in the study of Wu et al. [29] MMF might have suppressed up-regulation of MCP-1 expression in diabetic kidneys in rats mainly via suppression of macrophage infiltration. These data suggest that there is a possibility that use of calcineurin inhibitors like CsA and Tc together with MMF or everolimus in one combination could prevent an increase in concentration of KIM-1 (TIM-1) and MCP-1 in renal tissue. Although not statistically significant (in comparison to control dams), we have observed the lowest concentrations of three biomarkers in dams from CEG group. This is quite new observation as the impact of everolimus on the level of chosen biomarkers was not examined in studies so far. In a study of Kędzierska [8] MCP-1 concentration in rats treated with another mTOR inhibitor, rapamycin was lower than in control rats. Similar observation was noticed in mice treated with rapamycin [13]. Rapamycin also inhibited cytokine generation stronger than MMF [16].

Histological preparations made from rat kidney displayed lesions mostly within the kidney tubules. The more acidophilic PCT and more basophilic DCT were present in kidneys of rats from control and CEG group; opposite changes were found in kidneys of rats from CMG and TMG group what could indicate the altered physiology of the cells. These changes observed in light microscopy were accompanied by apoptotic changes found in nephron tubules. Apoptosis leads to the elimination of unwanted cells and abnormalities in this process can lead to the survival of pathological cells and their products, with further adverse consequences, such as the development of fibrosis. Dams from CMG and TMG group exhibited more pronounced apoptosis in the proximal nephron tubules compared to dams from control and CEG group. All treatment groups of dams showed more pronounced apoptosis in the distal tubules compared to control group. Data from previous studies show that CsA may induce renal tubular cell apoptosis through various mechanisms [30]. In experiments on rats after unilateral nephrectomy, where ischemia–reperfusion phenomenon was induced in the remnant kidney, a decrease in apoptosis and caspase-3 activity in proximal tubules of the groups treated with tacrolimus, rapamycin and MMF was observed. In the CsA treated group, apoptosis intensity was increased [31]. Kędzierska et al. [10] in a study conducted on male adult rats obtained results similar to ours—she has found more pronounced apoptosis in distal nephron tubules of rats treated with CsA; in rapamycin-treated rats the apoptosis was inhibited in the proximal tubules; in MMF treated rats intense apoptosis was observed in the proximal nephron tubules. She has also observed lower intensity of apoptosis in the distal tubules in Tc-treated rats what was not confirmed in our study.

Except of changes in tubules, a few collapsed glomeruli were visible within the cortex of rats from TMG group, possibly due to nephrotoxic effect of tacrolimus well described before [11].

In summary, all used immunosuppressive regimens in female Wistar rats before and during pregnancy caused abnormalities affecting mostly structure and function of nephron tubules. In all groups of treated dams alterations in serum ions were found. All combinations of immunosuppressants might induce changes visible in light microscopy (the early stages of kidney damage?) which were confirmed in more detailed evaluation (apoptosis assessment). It is probable that regimens containing calcineurin inhibitors and MMF are harmful to the kidney tubules, hence the increase in tubular cell apoptosis rate may be the evidence of their nephrotoxicity. Regimen based on mTOR inhibitor, everolimus had a lower rate of apoptosis observed in the proximal tubules. On the other hand in dams treated with everolimus despite a lower rate of apoptosis and lower kidney injury markers accumulation we observed higher serum creatinine concentration. One should remember that if apoptosis is inefficient, the repair processes may become abnormal, leading to pathological accumulation of cells and their products with further adverse consequences, such as the development of fibrosis. Thus interpretation which combination of drugs is better and which is worse for kidney morphology and long-lasting functioning in pregnant females is difficult and require further studies.
